# Buffalo Academy of Medicine

**Published:** 1900-01

**Authors:** J. C. Clemesha


### Society Proceedings.

### BUFFALO ACADEMY OF MEDICINE.

*Section on Medicine, Tuesday Evening, November 14, 1899.*

Reported by J. C. CLEMESHA, Secretary.

THE Medical Section of the Academy of Medicine held its second regular meeting in the Academy parlors, November 14, 1899. Meeting called to order at 8.30 p. m., Dr. E. H. Long in the chair.

The secretary referred to the use of salicylate of soda in insomnia, mentioning a case were it had been administered for another condition, but with happy effect on the sleeplessness of the patient. Dr. A. G. Bennett reported a case of furunculosis cured by large doses of soda salicylate, accidentally administered.

Dr. JACK, of Depew, then read a paper on The hygiene of the asthmatic. Referring to a previous paper on Asthma, read before the Academy, in which he described asthma as an "abnormal biochemy, due to altered conditions of the blood and divided into (1) anemic; (2) leukemic; (3) lithemic forms. He went on to consider the various methods of treatment. These he divided into diet, habits, baths, altitude, light and habitation. Dr. Jack advocated a gradual change to a proteid diet, with a free use of fruit rich in acids, as well as foods rich in alkalies. Hydrotherapy was next in importance to diet, while sunlight and a life at high altitudes, exercised a beneficent effect upon the disorder. He discountenanced the use of alcohol and tobacco, and noted that drug habits were often imposed upon asthmatics by physicians.

Dr. JOHN H. PRYOR then read a paper on Protracted pneumonia, with slow recovery. He had noticed the following groups: (1) Attack prolonged four to eight weeks, ending in recovery. Resolution gradual, protracted or incomplete; (2) relapsing or recurrent pneumonia: (3) subsidence of symptoms, but consolidation remaining and a later return of symptoms; final recovery; (4) pneumonia of a lobe or part of a lobe remaining three or four months; (5) pneumonia, assuming a chronic form and ending as "fibroid phthisis." In discussing the treatment of such cases Dr. Pryor laid great stress upon "respiratory exercise," with or without the pneumatic cabinet.

DISCUSSION OF DR. JACK'S PAPER.

Dr. BLAAUW thought asthma was due to a nervous cause and could not be cured by any one method of treatment.

DISCUSSION OF DR. PRYOR'S PAPER.

Dr. B. G. LONG quoted several cases from his practice where change of air and altitude had been of greatest benefit in chronic pneumonia.

Dr. JEWETT emphasised the use of exercise of the chest in such troubles and of the uselessness of cough mixtures, so-called

Meeting adjourned 10.05 p. m; members present, 33.

*Section on Medicine, Tuesday Evening, December 12, 1899.*

The third regular meeting of the Section of Medicine was held at the Academy Parlors, Market Arcade, December 12, 1899. Meeting called to order at 8.30 p. m., Dr. Eli H. Long in the chair. The minutes of the last meeting were read and approved.

Dr. A. L. Benedict presented a case of general splanchnoptosis, which he said, though not typical, showed a downward displacement of the liver, colon, stomach, left kidney; and commented on the rarity of liver displacements.

Dr. J. H. Dowd reported a case of thecitis due to gonorrhea. A man consulted him September 28, 1899, having had a gonorrheal discharge for two weeks. On October 17th he compained of pain in the back and right knee. On October 20th, pain had left the back and knee, but there was much pain in the flexors of the left femoral region (sartorius not included). Between this and November 3d, the pain persisted and moved to the popliteal space, at the same time involving an area over the sartorius muscle. On November 10th, the pain had disappeared, as had also the gonorrheal discharge. Dr. Dowd stated that Dr. Clemesha had called his attention to a case reported by Prof. Eichorst, which although being similar in complicating gonorrhea was accompanied by pronounced swelling of the region involved, and was diagnosticated as myocitis. His case, although involving the same region was not accompanied by swelling. Prof. Eichorst concludes that this complication always involves the femoral region, and is due to gonococci and not staphylococci.

Dr. M. D. Mann reported the case of a lady on whom he had done an Alexander's operation for backward displacement. She afterward married and became pregnant with a return of the backward displacement. On examination the retroversion was found due to the weight of an extremely dilated stomach lying on the enlarged and gravid uterus.

Dr. A. A. Jones and Dr. A. W. Bayliss reported severe cases of measles, in which prominent symptoms were, obstinate vomiting and diarrhea.

Dr. Rochester reported a series of cases among children of one family, probably of scarlatina sine enthemata. After short periods of slight illness, and without rash there was free desquamation of the epidermis.

Dr. A. A. Jones read a paper on The relation of indicanuria to the secretion of hydrochloric acid. He had based his paper upon the statement of Simon, of Baltimore, that the presence of indican in the urine was proof of the absence of free hydrocloric acid in the stomach.

In a series of 162 cases, Dr. Jones found that in 85 there was free hydrochloric acid with no indicanuria; in 36 there was free hydrochloric acid with indicanuria; in 25 there was no hydrochloric acid, no indicanuria; in 16 there was no free hydrochloric acid with indicanuria. Thus he believed that Simon's conculsions are wrong and quoted the researches of Vaux, of Ottawa, Canada, with whom he was more in agreement. Dr. Jones gave the history of indican in the urine (Prout, in 1840) its nature, formation and site and the clinical aspects of its presence in large quantities. Amongst various conditions it is present in intestinal putrefaction, ileus, stricture of small intestine, gastric ulcer.

Dr. A. L. Benedict in discussing the paper agreed with Dr. Jones in his conclusions and asked how far indican in the urine might be considered as a test of renal permeability?

Dr. Rochester related his experience, after perusal of Simon's paper, of giving hydrochloric acid in certain cases with indicanuria. He had to stop the use of the acid, and concluded that Simon's conclusions could not be taken as an index of treatment.

Dr. Stockton spoke briefly, congratulating Dr. Jones on his paper and referred to indicanuria present in achylia gastrica.

Dr. Dowd—I am sorry that Dr. Jones did not lay more stress on the fact that indican should be searched for in every case. Being associated as it is with putrefactive changes in the intestinal tract and also found in marked quantities in intestinal indigestion. I am sure that a correction of these would be not only very beneficial to the patient's condition but also aid us in the disease for which we may be treating them at the time. Simon says it is as important to search for indican as it is for albumin.

It was intended to take up the personal experience of members in regard to certain new drugs, but the lateness of the evening decided an adjournment.

Members present, 39.

*Section on Pathology, Tuesday Evening, November 21, 1899.*

Reported by FREDERICK H. MILLENER, M. D., Secretary.

THE third regular meeting of the Pathological section for the season, was held on Tuesday, November 21, 1899.

The meeting was called to order by the chairman, Dr. A. G. Bennett, at 8.50 p. m.

Forty-two Fellows were present.

The minutes of the preceding meeting were read and approved.

The subject for the evening was a paper by Dr. C. S. Butler, entitled, Pathological dentition. He said at the same time that changes are taking place in the teeth there are changes going on in the remote organs of the body, which have a marked bearing on the teething process. Preponderance of the spinal nervous system is especially marked in the infant, and it is owing to this fact that the sympathy of distant organs with one another is much greater in infancy than in adult life and produces in them a special tendency to reflex nervous disturbance. The teeth in infancy are made up largely of nerve tissue. At birth the child has not only twenty deciduous teeth advanced toward calcification, but also the germs of the twenty-four permanent teeth. It is plain to be seen that the future condition of the teeth may be jeopardised by any circumstance affecting the health of the mother during this period. At present it is the opinion of a vast majority of authorities that teething is frequently the only assignable cause of some of the diseases occurring during the dentition period. In several cases children suffering with fever, diarrhea, anuria, paralysis, and convulsions, were cured by lancing the gums, when other treatment had failed to relieve.

The paper was discussed by Doctors Irving M. Snow, H. R. Hopkins, Delancey Rochester, E. H. Long, Low and C. W. Stainton.

Dr. Irving M. Snow said that about the six or seventh month is the time of much sickness among children, and more die at that time than at any other time. He thought the vast majority of illness with children is due to other causes than dentition. He never saw a case of convulsion that was not excited by another cause.

Dr. H. R. Hopkins believed that dentition played an important rôle in causation of disease.

Dr. Delancey Rochester stated the case of a child of about eighteen months that had a nervous disturbance and cough followed by convulsions which remedies did not relieve, while lancing the child's gums caused in twenty-four hours the symptoms to subside.

Dr. E. H. LONG stated that he thought when either extreme was adopted in reference to the developmental disturbance of infancy, we were taking a fragmentary view. There are diseases which could not be assigned to teeth.

Dr. BUTLER closed the discussion by saying that more attention is given to the question of dentition as it relates to any period of life and to the general system in Europe than in this country. He related the case of a woman who went to a hospital in Hamburg to be treated for paralysis, and the first question she was asked was if her teeth had been examined.

---

*Obstetrical Section, Tuesday Evening, November 28, 1899.*

REPORTED BY ALBERT J. COLTON, M. D., Secretary.

A REGULAR meeting of the Obstetrical Section was held November 28, 1899, at the Academy Parlors. The chairman, Dr. H. D. INGRAHAM,, called the meeting to order at 8.45 p. m. The minutes of the previous meeting were read and approved.

The first paper of the evening, entitled, Lessons from practice in obstetrics, was read by Dr. P. W. VAN PEYMA.

The paper was filled with practical and instructive points, which the author had learned and gathered from his large obstetrical practice. He reviewed asepsis and antisepsis and expressed his opinion that absolute cleanliness was of much more importance than antiseptic washes and douches. He did not recommend antiseptic soaps, neither did he advise frequent douching after labor, especially if he had an inexperienced nurse. He disapproved of the early use of the forceps in labor and expressed his opinion that a patient in labor was safer in the hands of a midwife than an inexperienced physician.

He spoke of posterior positions and said that they occurred about once in five vertex presentations, and that fourteen out of fifteen would be converted into anterior positions if left to nature. He said in face presentations, if the chin was posterior, he let it progress and at last deliver with forceps; but if the chin was anterior, he attempted early to convert it into an anterior vertex position. He also spoke of placenta previa, and said where it was central he tamponed and waited for full dilatation before delivery. He said a weekly examination of the urine of a pregnant woman in the latter part of pregnancy was not sufficient and cited an example. The patient or some one in charge should be instructed if persistent headache occurred to notify the doctor at once. He laid great stress on diagnosis of position. Many other interesting and instructive points were mentioned.

The second paper of the evening was read by Dr. IRVING P. LYON, on the Mensuration and capacity of female bladder. The paper was very exhaustive, showing study and original research. Many original cuts and plates were shown to illustrate it. His study revealed that the rectum in a majority of the cases lies on the right side rather than on the left, as taught in text-books, also that the uterus in his examinations lies to the left of the median line. His method of measuring the capacity of the female bladder with air was quite novel and unique.

The papers were discussed by Drs. Mann, Grosvenor, Frederick, Rochester, Hopkins, James S. Smith, Jones and Stoddart.

Dr. MANN led in the discussion and disagreed with Dr. Lyon as to the air pressure of fifteen pounds to the square inch being the distending force while a patient was in knee-chest position, but that it depended on the posture of the patient, also that the distending pressure varies according to the individual. In regard to Dr. Van Peyma's paper he said that there were points enough to take a week to discuss. He said that his views were pretty well known, as he had threshed them over with Dr. Van Peyma many times. He disagreed with the author as to the non-use of antiseptic soaps for the hands. Where a physician was attending contagious and pus cases he recommended that a thin rubber cot be placed on the examining finger to prevent infection. He agreed with Dr. Van Peyma as to diagnosticating position in face presentations and believes that it should be corrected early. He, however, did not agree with him as to tamponing in placenta previa until there was full dilatation; but thought best to perform version by the Braxton Hicks method as soon as the hand could be introduced and bring down a leg to act as a tampon.

Dr ROCHESTER said in regard to eclampsia, where an attack comes on give morphia hypodermatically until the fits are controlled, and then get the patient to sweating by the use of hot air. If unsuccessful use small doses of pilocarpin in connection therewith. He has seen free perspiration produced with as small a dose as 1.50 of a grain. Watch the urine, especially as to reaction, as it is usually strongly acid in pregnant women. As to use of chloride of mercury as a uterine douche, he had employed it in his early practice as strong as 1-1000 without any ill-effects. He thought that where there was a rise of temperature due to decomposed, retained membranes that it was better to irrigate the uterus freely than to resort at once to the curette.

Dr. GROSVENOR said in regard to post partum hemorrhage that he had best results in controlling it by pouring cold water from a distance on the abdomen, directly over the uterus, also by introducing his closed hand into the uterus and making pressure from without until his fist was actually forced out by uterine contraction.

Dr. FREDERICK spoke particularly as to antisepsis of hands. Uses rubber gloves in making all examinations of patients, rectum, vagina, and the like, also in dressing pus cases, thus preventing the infecting of his hands and the danger of carrying infection to others. He said great caution should be used in changing from face to vertex presentations as he had caused rupture of uterus thereby.

Dr. HOPKINS thought the hands could be made obstetrically antiseptic though they could not be made germless.

Dr. JAMES S. SMITH thought the paper an admirable one and complimented its author.

Dr. STODDART asked if the torn ends brought together after a ruptured perineum held readily and firmly.

Dr. A. A. JONES asked Dr. Mann what kind of germs were on his son's hands after his experiments in making them aseptic, also if there was not a probability that he reinfected them in the air while passing from the antiseptic solutions to the culture media.

Dr. Mann said the germs found were not pathogenic.

The discussions were then closed by Drs. Van Peyma and Lyon.

There were present thirty-six members. Meeting adjourned at 10.45 p. m.

